# Ursolic acid inhibits the growth of human pancreatic cancer and enhances the antitumor potential of gemcitabine in an orthotopic mouse model through suppression of the inflammatory microenvironment

**DOI:** 10.18632/oncotarget.7537

**Published:** 2016-02-20

**Authors:** Sahdeo Prasad, Vivek R. Yadav, Bokyung Sung, Subash C. Gupta, Amit K. Tyagi, Bharat B. Aggarwal

**Affiliations:** ^1^ Department of Experimental Therapeutics, Cytokine Research Laboratory, The University of Texas MD Anderson Cancer Center, Houston, TX, USA; ^2^ Anti-inflammatory Research Institute, San Deigo, CA, USA

**Keywords:** ursolic acid, pancreatic cancer, NF-kappaB, anticancer, chemosensitization

## Abstract

The development of chemoresistance in human pancreatic cancer is one reason for the poor survival rate for patients with this cancer. Because multiple gene products are linked with chemoresistance, we investigated the ability of ursolic acid (UA) to sensitize pancreatic cancer cells to gemcitabine, a standard drug used for the treatment of pancreatic cancer. These investigations were done in AsPC-1, MIA PaCa-2, and Panc-28 cells and in nude mice orthotopically implanted with Panc-28 cells. *In vitro*, UA inhibited proliferation, induced apoptosis, suppressed NF-κB activation and its regulated proliferative, metastatic, and angiogenic proteins. UA (20 μM) also enhanced gemcitabine (200 nM)-induced apoptosis and suppressed the expression of NF-κB-regulated proteins. In the nude mouse model, oral administration of UA (250 mg/kg) suppressed tumor growth and enhanced the effect of gemcitabine (25 mg/kg). Furthermore, the combination of UA and gemcitabine suppressed the metastasis of cancer cells to distant organs such as liver and spleen. Immunohistochemical analysis showed that biomarkers of proliferation (Ki-67) and microvessel density (CD31) were suppressed by the combination of UA and gemcitabine. UA inhibited the activation of NF-κB and STAT3 and the expression of tumorigenic proteins regulated by these inflammatory transcription factors in tumor tissue. Furthermore, the combination of two agents decreased the expression of miR-29a, closely linked with tumorigenesis, in the tumor tissue. UA was found to be bioavailable in animal serum and tumor tissue. These results suggest that UA can inhibit the growth of human pancreatic tumors and sensitize them to gemcitabine by suppressing inflammatory biomarkers linked to proliferation, invasion, angiogenesis, and metastasis.

## INTRODUCTION

Carcinoma of the pancreas has had a markedly increased incidence during the past several decades. Pancreatic cancer accounts for about 7% of all cancer deaths and ranks fourth as a cause of cancer death among both men and women in the US. In 2015, an estimated 48,960 new cases and 40,560 deaths from pancreatic cancer will occur in the United States [1]. High rate of mortality is associated with pancreatic cancer, however the cause of a disease or condition is poorly understood [2]. This cancer is rarely curable and has an overall survival rate of less than 4% [3]. Although surgery, chemotherapy, radiation therapy, immunotherapy, and vaccine therapy are used for the treatment of pancreatic cancer, these therapies are less effective than in some other and highly expensive.

Gemcitabine is the standard chemotherapeutic agent for advanced pancreatic cancer. Clinical trials of various phases have investigated combinations of gemcitabine with erlotinib [an epidermal growth factor receptor (EGFR) inhibitor], platinum analogues, bevacizumab [a vascular endothelial growth factor (VEGF) inhibitor], or celecoxib [a cyclooxygenase-2 (COX-2) inhibitor] [4]. However, the objective tumor response rate to gemcitabine is less than 10%, and the drug offers only a marginal survival benefit [5]. Furthermore, gemcitabine is associated with the development of drug resistance. Thus, novel agents with minimum toxicity and ability to sensitize pancreatic cancer cells to gemcitabine are highly needed.

One compound with potential for this use is ursolic acid (UA), a pentacyclic triterpenoid that has been identified in a large variety of medicinal plants, including rosemary and holy basil. Earlier studies have shown that UA can inhibit proliferation and induce apoptosis in leukemia, melanoma, and cancers of the breast, prostate, lung, and endometrium [6–10]. UA has been shown to inhibit tumor progression [11, 12], to induce tumor cell differentiation [13], and to exhibit antiangiogenic activity [14]. UA also has been shown to modulate multiple cancer-related signaling mechanisms; for example, UA inhibits DNA replication [15, 16], activates caspases [7, 17] and c-Jun N-terminal kinases (JNK) [18], downregulates antiapoptotic genes [19, 20], inhibits COX-2 and inducible NO synthase expression [21, 22], suppresses matrix metallopeptidase (MMP)-9 [23], and inhibits protein tyrosine kinase [15]. Previous work from our laboratory has shown that UA can inhibit signal transducer and activator of transcription 3 (STAT3) [24] and nuclear factor (NF)-κB activity [19] and can induce apoptosis in cancer cells by upregulating death receptors [25]. Since reactive oxygen species (ROS) requires introduction of a therapeutic agent for maximal effect [26], UA induce apoptosis of cancer cells through ROS generation [25]. In animal models, UA was found to be chemopreventive [11, 27, 28]. In other animal studies, UA suppressed tumor invasion [23], sensitized orthotopically implanted colorectal tumors to capecitabine [29], and inhibited the experimental metastasis of esophageal carcinoma [30]. However, whether UA has antitumor potential against pancreatic cancer is not known.

In the present study, we investigated whether UA can enhance the effect of gemcitabine against human pancreatic cancer cells *in vitro* and in orthotopically implanted nude mice. Whether the inhibition in tumor growth is connected with changes in the expression level of miR-29a, a member of miR-29 family, was investigated [31]. We found that UA inhibited proliferation of various human pancreatic cancer cells and potentiated the antitumor activity of gemcitabine through the inhibition of transcription factors NF-κB and STAT3 as well as multiple inflammatory gene products regulated by NF-κB and STAT3. Both UA and gemcitabine suppressed the expression of miR-29a and the expression was further suppressed by the combination of two agents.

## RESULTS

The aim of the present study was to determine whether UA could improve the efficacy of gemcitabine against pancreatic cancer. To determine this, the mechanism by which UA manifests its effects was investigated against human pancreatic cancer cells *in vitro* and in an orthotopic nude mouse model.

### UA inhibits proliferation and induces apoptosis of pancreatic cancer cells *in vitro*

**Figure 1:**
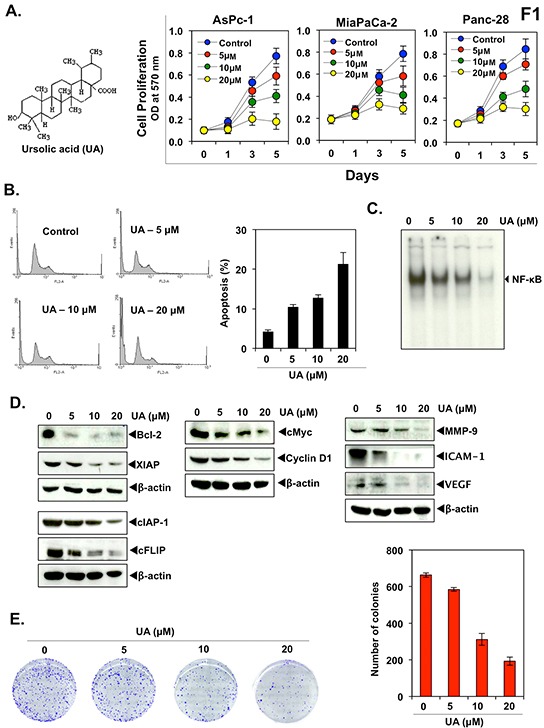
Ursolic acid (UA) inhibits pancreatic cancer cell growth and proliferation and enhances the apoptotic effects of gemcitabine **A.** Chemical structure of UA (left panel). AsPC-1, MIA PaCa-2, and Panc-28 (2000 cells/well) cells were treated with indicated concentration of UA for 1, 3, and 5 days. MTT analysis was performed as indicated in Materials and Methods (right panel). **B.** Panc-28 (1×10^6^ /well) cells were treated with indicated concentration of UA for 24 hours. Apoptosis was determined by the FACS analysis, measuring sub-G1 peak as apoptosis indicator. **C.** Panc-28 (1×10^6^) cells were treated with UA for 8 hours. Nuclear extract were prepared and DNA binding essay was done by EMSA. **D.** Panc-28 (1×10^6^) cells were treated with UA with indicated concentration for 24 hours. Whole cell extract were prepared and subjected to western blot. **E.** Panc-28 (1,000 cells/well) were treated with UA with indicated concentration. After 12 hours medium was replaced with fresh medium and incubated for 9 days. Cells were stained with crystal violet and counted for colony formation.

To determine whether UA inhibits proliferation in human pancreatic cancer cells, we treated AsPC-1, MIA PaCa-2, and Panc-28 cells with 0, 5, 10 and 20 μM UA for 1, 3, or 5 days and measured cell viability using an MTT assay. UA inhibited proliferation in all 3 cell lines in a dose- and time-dependent manner (Figure 1A, right panel).

Next, we determined whether UA induces apoptosis of pancreatic cancer cells. We treated the pancreatic cancer Panc-28 cells for 24 hours analyzed by FACS for apoptosis. We found that UA exhibited dose dependent apoptosis of pancreatic cancer cells from 4.17% in control to 21.23% in UA (20 μM) treated cells (Figure 1B).

### UA suppresses constitutive NF-κB activation and its regulated gene products in pancreatic cancer cells

Because NF-κB has been linked with both proliferation and chemoresistance, we next examined whether UA could inhibit constitutive NF-κB activation in Panc-28 cells. Our results showed that UA inhibited constitutive NF-κB activation in a dose-dependent manner (Figure 1C).

Next we determined whether UA also suppresses NF-κB regulated gene products. Panc-28 cells were exposed to UA at different concentration and then analyzed by western blotting. Results showed that UA dose-dependently inhibited the expression of proteins linked with survival, proliferation, invasion and metastasis (Figure 1D and Supplementary Figure S1).

### UA inhibits colony formation ability of pancreatic cancer cells

We also examined whether UA affects the long-term colony formation ability of pancreatic cancer cells, which more closely mirrors the situation *in vivo*. As shown in Figure 1E, UA dose-dependently inhibited colony formation of Panc-28 cells. At the dose of 20 μM UA decreased 74.2% number of colonies compared to untreated control.

### UA enhances cytotoxic effects of gemcitabine

**Figure 2:**
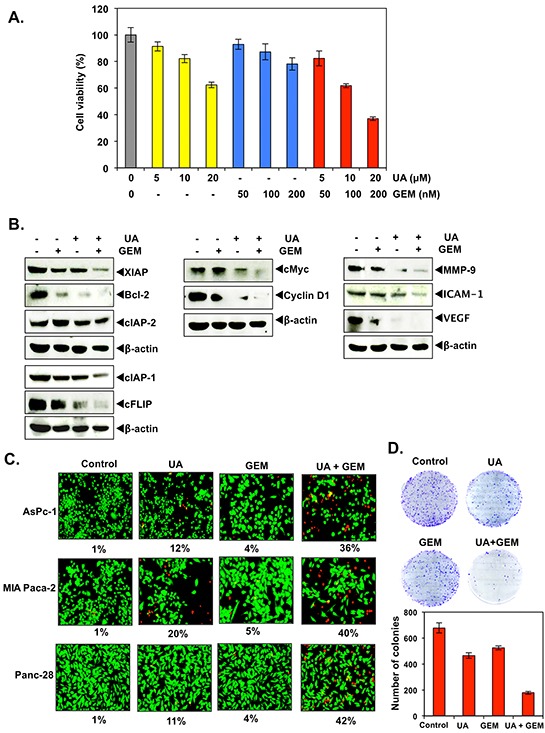
**A.** Panc-28 (5,000/well) cells were treated with indicated concentration of UA for 8 hours. Cells were washed with PBS to remove UA and then exposed with different concentration of gemcitabine for 24 hours. Cytotoxicity was determined by MTT assay as described in Materials and Methods. **B.** Panc-28 (1×10^6^) cells were treated with UA (20 μM) for 8 hours. Cells were washed with PBS to remove UA and then exposed with gemcitabine (200 nM) for 24 hours. Whole cell extract were prepared and subjected to western blot. **C.** Panc-28 (5,000 cells/well) cells were treated with UA (20 μM) for 8 hours. Cells were washed with PBS to remove UA and then exposed with gemcitabine (200 nM) for 24 hours. A LIVE/DEAD assay was performed as per the instruction of manufacturer. The percentages of dead cells (red) are shown. **D.** Panc-28 (1,000 cells/well) cells were treated with UA (20 μM) for 8 hours. Cells were washed with PBS to remove UA and then treated with gemcitabine (200 nM) for 12 hours. Medium was replaced and incubated for 9 days. Cells were stained with crystal violet and counted for colony formation.

To determine whether UA not only induces cytotoxicity rather it enhances the inhibitory effects of gemcitabine, pancreatic cancer cells were treated with different concentration of UA or gemicitabine alone or in combination. Results obtained from MTT assay showed that UA or gemicitabine alone induced moderate cytotoxicity in dose dependent manner. However, it also increased the cytotoxic activity of gemcitabine dose dependently (Figure 2A).

To evaluate the type of drug interactions, we calculated the combination index (CI) values using CalcuSyn software based on the median effect principle [32]. The obtained CI values demonstrated the positive interactions between UA and gemcitabine, and synergy was observed for all doses of UA and gemcitabine combinations in pancreatic cancer cells. The combination ratio of UA and gemcitabine (1:0.01) resulted in a potent synergistic profile (CI values between 0.46, 0.51, 0.56 and 0.59) at calculated ED50, ED75, ED90, and ED95. Thus, we demonstrated that UA is highly efficient in combination with gemcitabine as an anticancer agent against pancreatic cancer cells in our *in vitro* models.

### UA increases the effect of gemcitabine in inhibition of cell survival, proliferative and metastatic proteins

To determine whether UA enhances the effects of gemcitabine in inhibition of cell survival, proliferative and metastatic proteins, Panc-28 cells were exposed to UA and then treated with gemcitabine. Western blot analysis showed that UA inhibited the expression of proteins associated with survival (XIAP, Bcl-2, cIAP-1, cIAP-2 and cFLIP), proliferation (cyclin D1 and cMyc), and invasion and metastasis (ICAM-1, MMP-9 and VEGF) were moderately inhibited by either UA or gemcitabine alone, however it enhanced the inhibitory effects of gemcitabine. The expression levels of cIAP-2, cMyc and ICAM-1 were not affected by gemcitabine but moderately or slightly decreased by UA treatment; however, their expression levels were decreased substantially by the combination of UA and gemcitabine (Figure 2B and Supplementary Figure S2).

### UA potentiates the apoptotic effects of gemcitabine, and inhibits colony formation ability of pancreatic cancer cells

To determine whether UA enhances gemcitabine-induced cell death, we pretreated AsPC-1, MIA PaCa-2, and Panc-28 pancreatic cells with UA and then gemcitabine. The LIVE/DEAD assay showed that UA and gemcitabine were highly effective at doses at which UA or gemcitabine alone were minimally effective (Figure 2C).

We also examined whether UA enhances the inhibitory effect of gemcitabine on long-term colony formation assay. UA or gemcitabine when administered alone had little effect on the colony-forming ability of Panc-28 cells. UA and gemcitabine alone had 30.3% and 21.5% decreased colony formation respectively compared to control. However, administration of UA and gemcitabine in combination significantly decreased (73.8%) the colony formation of Panc-28 cells (Figure 2D).

### UA inhibits the growth of orthotopically implanted pancreatic cancer in nude mice

**Figure 3:**
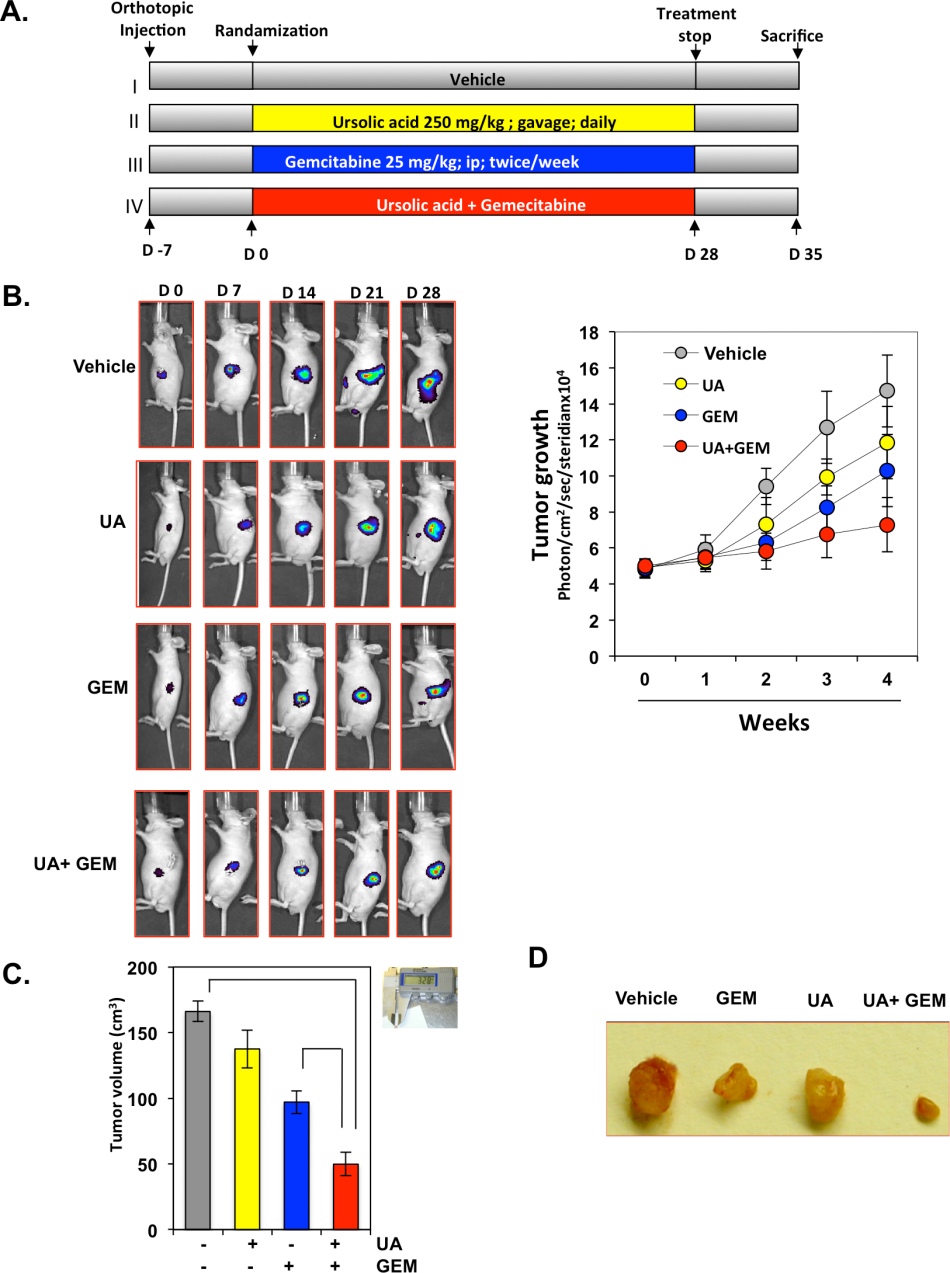
UA enhances the effect of gemcitabine (GEM) to inhibit the growth of orthotopically implanted pancreatic cancer tumors in nude mice **A.** Schematic representation of the experimental protocol described in the “Materials and Methods” section. Mice were randomly assigned to 4 treatment groups (n=10): group I was given corn oil (100 μL, orally, daily); group II was given UA (250 mg/kg orally, daily); group III was given gemcitabine twice per week (25 mg/kg, intraperitoneally, twice a week); and group IV was given UA (250 mg/kg orally, daily) and gemcitabine (25 mg/kg, intraperitoneally, twice a week). **B.** Bioluminescence imaging of orthotopically implanted pancreatic tumors in live, anesthetized mice was performed every week (left panel). Measurements (photons/sec) of mean tumor volume on bioluminescence imaging at various time points are shown (right panel). **C.** Mean tumor volumes measured on the last day of the experiment at autopsy using Vernier calipers and calculated using the formula V = 2/3πr^3^. **D.** Photographs of mice and tumors from each treatment group taken at autopsy.

Figure 3A depicts the experimental protocol we used to evaluate the effects of UA and gemcitabine alone and in combination on the growth of orthotopically implanted human pancreatic cells in nude mice. We decided to use Panc-28 cells for *in vivo* studies because this cell line is stably transfected with luciferase.

The bioluminescence imaging (Figure 3B, left panel) results showed that the gradual increase in tumor volume was greater in the vehicle-treated control group than in the other treatment groups (Figure 3B, right panel). The mean tumor volume in the group treated with the combination of UA and gemcitabine was significantly lower than the tumor volumes in the groups treated with UA alone or gemcitabine alone.

We found that treatment with UA alone inhibited tumor growth compared with controls (Figure 3C). Treatment with gemcitabine alone was effective in suppressing 41.6% tumor growth compared with controls and was more effective than treatment with UA alone (17.2%). The combination of the two agents had prominent efficacy (70% compared to control) in reducing the tumor burden than was either agent alone. The final mean tumor volume in the group treated with the combination of UA and gemcitabine was significantly lower than the tumor volumes in the groups treated with UA alone or gemcitabine alone (Figure 3D).

### UA inhibits distant organ metastasis from orthotopically implanted pancreatic cancer in nude mice

**Figure 4:**
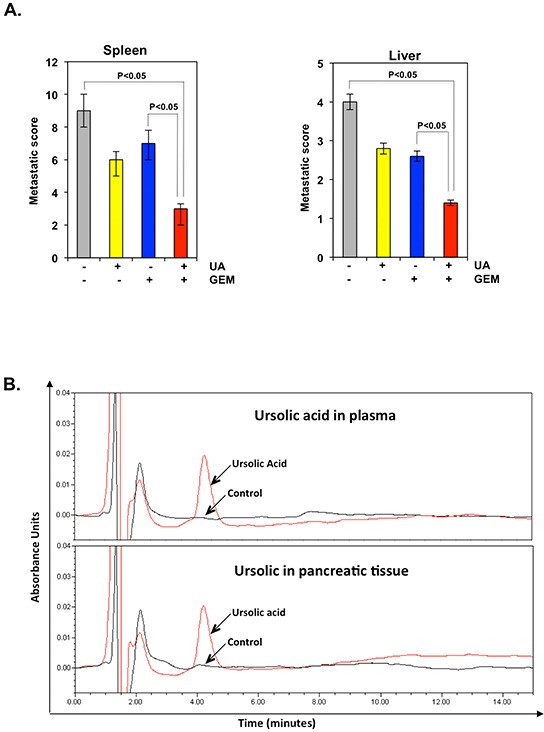
**A.** UA and gemcitabine (GEM) inhibit distant organ metastasis from orthotopically implanted pancreatic cancer in nude mice. UA and gemcitabine combined inhibited metastasis to the spleen and liver. **B.** UA is bioavailable in the serum of nude mice and in pancreatic cancer tumors. Animals were fed with UA 4 h before being sacrificed. Mice were anesthetized and blood was collected by cardiac puncture, and then the mice were sacrificed humanely. HPLC analysis was performed to determine bioavailability of UA in the serum and pancreatic cancer tumors.

At autopsy, the mice in each treatment group were examined for the presence of metastases. The results showed that pancreatic cancer metastasis developed more frequently in the spleen and liver of vehicle-treated mice than in mice treated with UA or gemcitabine alone. UA and gemcitabine respectively decreased to 33.3% and 22.2% metastasis of pancreatic cancer cells in spleen while in liver it decreased to 30% and 35%. Maximum inhibition of metastasis, however, was observed in the group treated with the combination of UA and gemcitabine 66.6% in spleen and 65% in liver (Figure 4A).

### UA is bioavailable in serum and tumor tissue

Because UA inhibited tumor growth in the mice, we investigated whether UA is bioavailable in the serum and in the pancreatic tumors of the mice. HPLC results showed that a significant amount of UA was available in the serum (442±35 ng/mL) and in the tumors (228±24 ng/g) of the mice treated with UA (Figure 4B). Although the amount of UA in the tumor tissue was less than that in the serum of mice, the two were similar.

### UA modulates cell proliferation and angiogenesis markers

**Figure 5:**
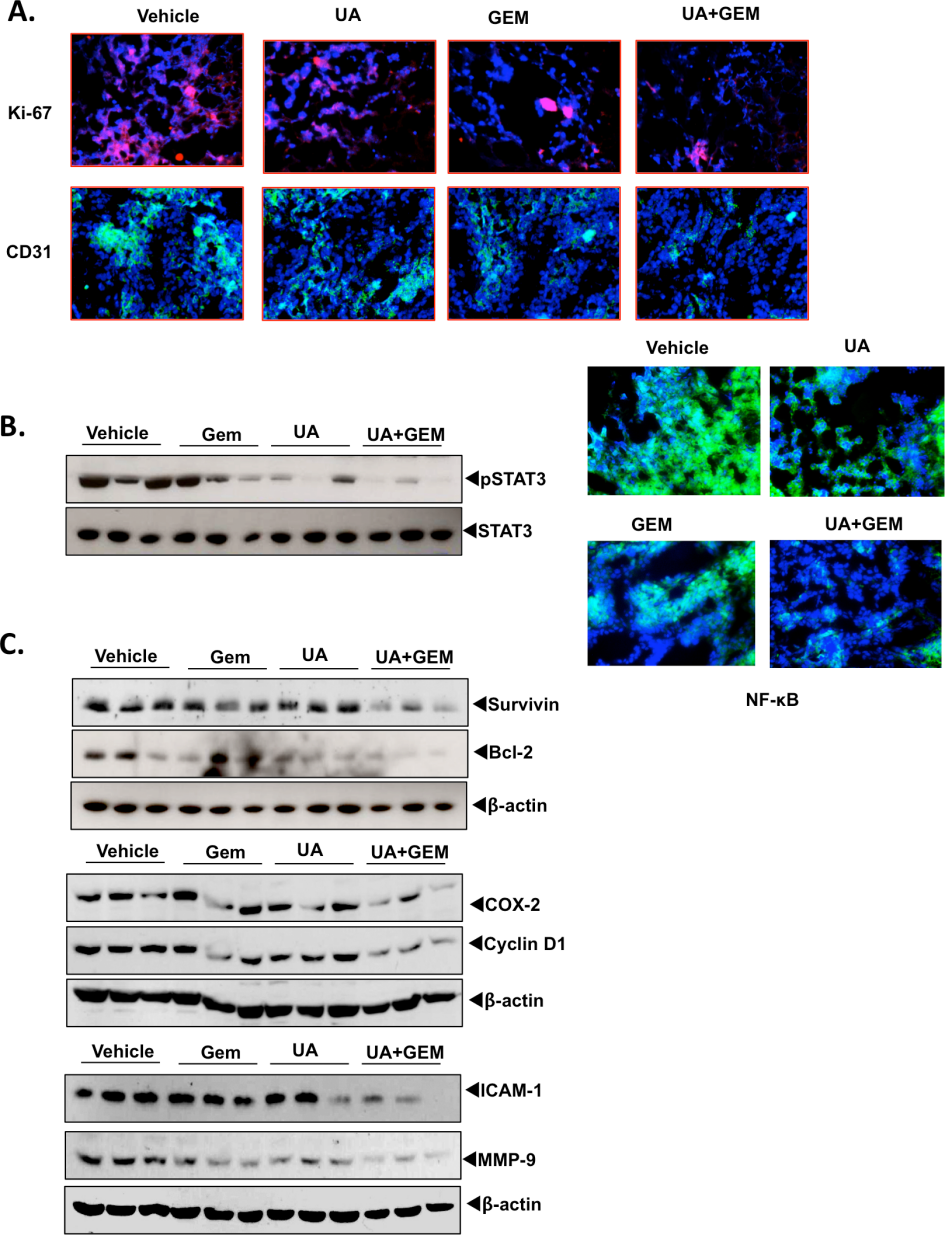
**B.** UA enhances the effect of gemecitabine against tumor cell proliferation angiogenesis and transcription factors activation in pancreatic cancer. **A.** Immunofluorescence analysis of proliferation marker Ki-67 and microvessel density CD31 was performed in tumor tissues. Samples from 3 animals in each group were analyzed by immunofluorescence, and representative data are shown. (B) UA inhibits STAT3 and NF-κB and the expression of their regulated gene products in orthotopically implanted pancreatic tumors. Western blot analysis showed that UA in combination with gemcitabine inhibited the activation of STAT3 (left). Immunofluorescence analysis of nuclear p65 showed the inhibition of NF-κB by UA alone and in combination with gemcitabine (right). **C.** Western blot analysis showed the suppression of proteins involved in cell survival, inflammation, proliferation, and metastasis by UA and UA combined with gemcitabine.

To determine whether UA affects pancreatic cancer growth and metastasis by modulating Ki-67 and CD31, we examined the expression of these markers of proliferation and angiogenesis, respectively. Our results showed that UA alone significantly decreased the expression of Ki-67 and CD31, but UA with gemcitabine provided the largest decrease in the expression of either marker (Figure 5A).

### UA inhibits the activation of transcription factors STAT3 and NF-κB in orthotopically implanted pancreatic tumors

Next examined whether UA modulates the STAT3 phosphorylation in pancreatic tumors. Western blot analysis showed that UA alone significantly suppressed the activated STAT3 by inhibiting phosphorylation at Tyr705, and the combination of UA and gemcitabine further enhanced the inhibitory activity (Figure 5B, left). Thus, inhibition of this transcription factor by UA could be associated with the enhancement of gemcitabine-induced pancreatic tumor regression.

We also investigated whether the effects of UA on tumor growth in the mice were associated with the inhibition of NF-κB activation. The results of Immunofluorescence analysis showed that treatment with UA alone and UA plus gemcitabine significantly suppressed NF-κB activation in tumor samples (Figure 5B, right).

### UA inhibits the expression of biomarkers linked to tumor survival, proliferation, and metastasis in orthotopically implanted pancreatic tumors

Next we examined whether UA modulates the expression of proteins involved in cell survival, invasion, and metastasis in orthotopically implanted pancreatic tumors. Western blotting revealed significant reductions in the expression of markers for cell survival (survivin and Bcl-2), proliferation (cyclin D1), inflammation (COX-2), and metastasis (ICAM-1 and MMP-9) in tumors from the groups treated with UA or gemcitabine alone compared with those from the control group. However, gemcitabine in combination with UA was more effective in suppressing the expression of these proteins than either UA alone or gemcitabine alone (Figure 5C).

**Figure 6:**
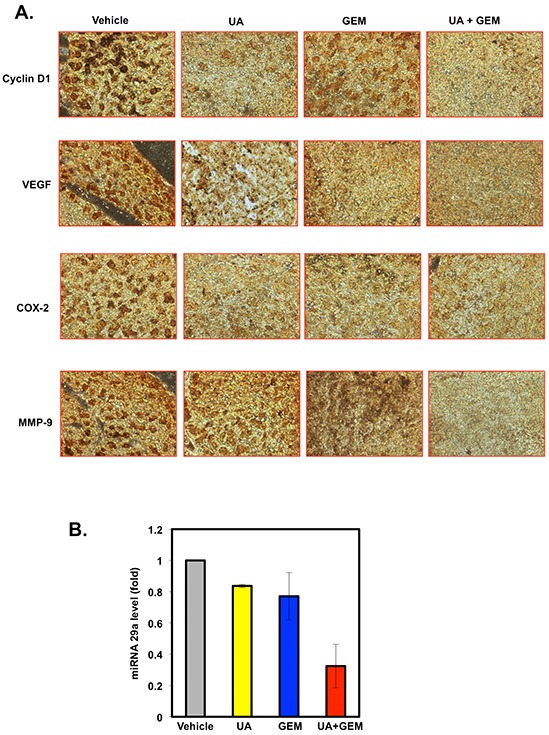
**A.** UA enhances the effect of gemcitabine (GEM) against the expression of cyclin D1, VEGF, COX-2, and MMP-9 in orthotopically implanted pancreatic tumor samples. **B.** Combination of UA and gemcitabine suppresses the expression of miR-29a in tumor tissues. Total RNA was extracted from the tumor tissues of normal and treated mice and examined for miR-29a expression level by qRT-PCR.

Immunohistochemical analysis of pancreatic tumor tissues also revealed that UA, alone or in combination with gemcitabine, significantly decreased the expression of cyclin D1, COX-2, VEGF, and MMP-9 molecules compared with the control treatment (Figure 6A).

Because miR-29a has been shown to induce resistance to gemcitabine in pancreatic cancer cells [33], we sought to investigate miR-29a expression in tumors from normal and treated mice. Results indicated that the expression of miR-29a was suppressed by 0.16 folds in gemicitabine group, by 0.23 folds in UA group, and by 0.68 folds in the combination group (Figure 6B).

## DISCUSSION

Currently, gemcitabine is the key chemotherapy for pancreatic cancer, however the drug has only 5.4% partial response rate and imparts a progression-free survival from 0.9 to 4.2 months [5]. Although patients with pancreatic cancer show initial sensitivity to gemcitabine, development of resistance is common. Thus, the agents that can replace or overcome the resistance to gemcitabine are needed. In the present study, we have shown that UA, a triterpene present in numerous dietary sources, can sensitize pancreatic cancer cells to gemcitabine. We found that UA inhibited proliferation in various pancreatic cancer cell lines, inhibited constitutive NF-κB activation, and potentiated the apoptosis induced by gemcitabine. Further, in an orthotopic nude mice model, we demonstrated that UA enhanced the antitumor effects of gemcitabine. UA, alone and in combination with gemcitabine, significantly suppressed gene products involved in cell survival, proliferation, and metastasis in orthotopically implanted pancreatic tumor tissue. In agreement with our observation that UA inhibited proliferation in pancreatic cancer cells, 2-substituted analogues of UA were found to inhibit proliferation in Panc-1 and Panc-28 cells [34].

In the present study, we found that gemcitabine alone induced apoptosis in MIA PaCa-2, AsPC-1, and Panc-28 cell lines minimally but that gemcitabine-induced apoptosis was significantly enhanced by UA. Similar to these results, another report showed that pancreatic cancer cells BxPC-3, Capan-1, and PancTu-1 were unaffected by gemcitabine treatment [35], likely due to the overexpression of NF-κB by these cells. The suppression of NF-κB activity may be a mechanism for the sensitization of pancreatic cancer cells to gemcitabine by UA. Supporting this hypothesis is a previous study in which prolonged treatment with relatively low doses of UA sensitized human lung and cervical cancer cells to paclitaxel or cisplatin through the suppression of NF-κB [36].

We also found that UA downregulated the expression of proteins associated with cell proliferation (cyclin D1 and COX-2) and survival (survivin, XIAP, cFLIP, Bcl-2, and cIAP-1), and this downregulation was enhanced when UA was given in combination with gemcitabine. Because these tumorigenic proteins are regulated by NF-κB and STAT3, it is likely that the inhibition of these transcription factors contributes to the downregulation of the proteins. The miR-29a family of microRNAs is aberrantly expressed in numerous cancer types [31]. A recent report indicated that miR-29a can induce resistance to gemcitabine in pancreatic cancer cells [33]. It is possible that the down-regulation in the expression of miR-29a by the combination of two agents might lead to the inhibition in NF-κB activation that in turn can lead suppression in the tumor growth. However, whether UA directly targets to miR-29a promoter or through mediation of other transcription factors remains to be elucidated.

In our orthotopic mouse model, UA or gemcitabine alone were equally potent in inhibiting tumor growth, but the combination of UA and gemcitabine further suppressed the tumor growth. In agreement with these observations, a previous study showed that UA inhibited the growth of DU145 cancer xenografts in nude mice [37]. The dose of UA used in this study was based on our previous study, where UA at 250 mg/kg was effective in chemsensitization of colorectal cancer. Besides these, other researchers have also used almost equal to this doses in animal model of different cancers [37, 38].

Our study, however, is the first to show that UA alone can inhibit pancreatic cancer metastasis to distant organs such as the spleen and liver. Our study also is the first of its kind to demonstrate that metastatic spread of pancreatic cancer is further suppressed by the combination of UA and gemcitabine. Further study showed that UA inhibited proteins involved in metastasis and angiogenesis, such as MMP-9 and VEGF. Suppression of these proteins could be the mechanism by which by UA and gemcitabine inhibited the metastasis of pancreatic cancer to the distant organs.

Next we examined the mechanism by which UA manifests its effects against pancreatic cancer in nude mice model and found that the proliferation marker Ki-67 as well as micro vessel density indicator CD31 was down regulated by UA. The downregulation of NF-κB activity in tumor tissues may also account for inhibitory effects of UA on tumor growth in mice model. The downregulation in the expression of tumorigenic proteins such as cyclin D1, COX-2, and survivin in tumor tissue may be due to the suppression of NF-κB activity. Previously we have shown that UA inhibited the expression of cell survival proteins survivin and Bcl-2 in a model of orthotopically transplanted human colorectal cancer in nude mice [29], which supports the findings of our current study.

One of the major concerns in cancer drug development is that most drugs possess limited bioavailability. However, in this study, we found a significant presence of UA in the pancreatic tumor tissue of mice. The bioavailability of *Oldenlandia diffusa*, one of the sources of UA, has also been shown in Caco-2 colon cancer cells [39]. The bioavailability of UA was also reported in the livers of mice after intravenous injection [40]. These studies further support the observations of bioavailability in the current study.

Our finding that UA enhanced the antitumor effect of gemcitabine in an animal model by inhibiting NF-κB and its downstream targets—leading to the inhibition of proliferation, angiogenesis, and invasion—suggests that UA has significant potential for the treatment of pancreatic cancer. Whether the synthetic triterpenoid C-28 methyl ester of 2-cyano-3,12-dioxooleana-1,9,-dien-28-oic acid (CDDO-Me; bardoxolone methyl)—which is a homologue of the UA family of compounds, is known to suppress inflammatory pathways [41], and is in clinical trial [42–44]—exhibits chemosensitization activities against human pancreatic cancer similar to those described here should be explored in the future.

As the doses of gemcitabine (25 mg/kg) and UA (250 mg/kg) used in our animal study are safe and relevant to that in human subjects, the current study provides a basis for evaluating the efficacy of gemcitabine and UA combinations in clinical trials.

## MATERIALS AND METHODS

### Reagents

UA was kindly supplied by Kingsing Guan, China. The antibodies against Bcl-2, COX-2, cyclin D1, ICAM-1, MMP-9, NF-κB p65, cIAP-1, pSTAT3, and STAT3 were purchased from Santa Cruz Biotechnology (Santa Cruz, CA); the antibodies against VEGF and Ki-67 (rabbit monoclonal clone SP6) were obtained from Neomarkers (Fremont, CA); the antibodies against survivin were obtained from R&D Systems (Minneapolis, MN); the antibodies against XIAP and cFLIP were obtained from Imgenex Corp (San Diego, CA) and the antibodies against β-actin were procured from Sigma (St. Louis, MO). Mouse CD31 monoclonal antibody was obtained from Pharmingen (San Diego, CA). The liquid DAB+ substrate chromogen system-HRP used for immunohistochemistry was obtained from Dako (Carpinteria, CA). Penicillin, streptomycin, RPMI 1640, and DMEM were obtained from Mediatech Inc. (Herndon, VA). Fetal bovine serum (FBS) was purchased from Atlanta Biologicals (Norcross, GA). Gemcitabine (Gemzar; Eli Lilly, Indianapolis, IN) was stored at 4°C and dissolved in sterile PBS on the day of use. D-luciferin potassium salt (Xenogen, Hopkinton, MA) was dissolved in sterile PBS at a concentration of 40 mg/mL.

### Cell lines

The human pancreatic cancer cell lines AsPC-1 and MIA PaCa-2 were obtained from the American Type Culture Collection (Manassas, VA). The Panc-28 human pancreatic carcinoma cell line was kindly provided by Dr. Shrikanth Reddy (The University of Texas MD Anderson Cancer Center, Houston, TX). MIA PaCa-2 and Panc-28 cells were cultured in DMEM, and AsPC-1 cells were cultured in RPMI 1640 supplemented with 10% FBS, 100 U/mL of penicillin, and 100 μg/mL of streptomycin.

### Proliferation assay

The effect of UA on cell proliferation was determined by the MTT uptake method as described previously [45]. Briefly, pancreatic cancer cells (2,000 per well) were treated with UA in quadruplicate in a 96-well plate and then incubated for 1, 3, or 5 days at 37°C. After addition of MTT solution to each well, cells were incubated for another 2 hours at 37°C. The lysis buffer (20% SDS and 50% dimethylformamide) was then added, and the cells were incubated overnight at 37°C. An MRX Revelation 96-well multiscanner (Dynex Technologies, Chantilly, VA) was used to measure the absorbance of the cell suspension at 570 nm).

### Propidium iodide (PI) staining for determination of apoptosis

The sub-G1 population of cells as indicator of apoptosis was analyzed by using PI staining as described previously [25]. The assay was performed with a BD FACSCalibur flow cytometer (BD Biosciences, San Jose, CA). A total of 20,000 events were analyzed by flow cytometry using an excitation wavelength set at 488 nm and emission set at 610 nm. Cells undergoing apoptosis lose part of their DNA (due to DNA fragmentation), which are detected as a sub-G1 population after PI staining.

### Clonogenic assay

The clonogenic assay determines the ability of cells in a given population to undergo unlimited division and form colonies. Panc-28 cells (1,000 per well) were seeded in 6-well plates, incubated for 12 hours, and then treated with different concentration of UA or gemcitabine. After 24 hours medium was replaced with fresh medium and allowed to form colonies. After 9 days, colonies were washed with PBS, fixed in a solution of methanol and acetic acid (3:1), stained with 0.25% crystal violet and counted manually.

### Cell death assay

To investigate whether UA could potentiate the efficacy of gemcitabine for causing apoptosis in pancreatic cancer cells, we used a LIVE/DEAD cell viability assay kit (Invitrogen), which is used to determine intracellular esterase activity and plasma membrane integrity and was described previously. AsPC-1, MIA PaCa-2, and Panc-28 cells (5,000 per well) were incubated in chamber slides, pretreated with 20 μM of UA for 8 hours. Cell were washed with PBS to remove UA and then treated with 100 nM of gemcitabine for 24 hours. The cells were then stained with the assay reagents for 30 minutes at room temperature, and cell viability was determined by counting live (green) and dead (red) cells under a fluorescence microscope.

### NF-κB activation in pancreatic cancer cells

To determine the level of NF-κB activation, we prepared nuclear extract from treated and untreated Panc-28 pancreatic cancer cells and carried out electrophoretic mobility shift assays essentially as described previously [46].

### Animals

Four-week-old male athymic *nu/nu* mice were obtained from the breeding colony of the Department of Experimental Radiation Oncology at MD Anderson. The animals were housed in standard poly(methyl methacrylate) cages (5 per cage) in a room maintained at constant temperature and humidity and in a 12-hour light/12-hour dark cycle. Their diet consisted of regular autoclave chow and water ad libitum. None of the mice exhibited any lesions, and all mice were tested pathogen free. All the mice were acclimatized to a pulverized diet for 3 days before orthotopic cancer cell implantation. This experimental protocol was reviewed and approved by the Institutional Animal Care and Use Committee at The University of Texas MD Anderson Cancer Center.

### Orthotopic implantation of Panc-28 cells

Panc-28 cells, stably transfected with luciferase, were orthotopically implanted in the pancreas of nude mice as described previously [45]. After anesthetizing the mice with ketamine-xylazine solution, a small incision was made in the left abdominal flank, and luciferase transfected Panc-28 cells (1 × 10^6^) in 50 μL PBS were injected into the subcapsular region of the pancreas with a 27-gauge needle. A cotton swab was held for 1 minute over the site of injection to prevent leakage. The abdominal wound was closed with wound clips (Braintree Scientific, Braintree, MA).

### Experimental protocol

One week after orthotopic implantation, 10 mice each were randomly assigned to the following treatment groups: (a) untreated control (corn oil, 100 μL daily orally), (b) UA alone (250 mg/kg daily orally), (c) gemcitabine alone (25 mg/kg twice weekly by intraperitoneal injection), or (d) UA (250 mg/kg daily orally) plus gemcitabine (25 mg/kg twice weekly by intraperitoneal injection).

Tumor volumes were monitored weekly with the IVIS 200 *in vivo* bioluminescence imaging system (Xenogen, Cranbury, NJ), which includes a cryogenic cooling unit and a data acquisition computer that uses Living Image software. Imaging was performed on days 0, 7, 14, 21, and 28 of treatment as previously described.

Therapy was continued for 4 weeks. One week after treatment ended, the mice were euthanized, and the primary tumors in the pancreas were excised. The final tumor volume was measured as V = 2/3πr^3^, where r is the mean of the three dimensions (length, width, and depth). Each tumor was divided in two. Half of the tumor tissue was fixed in formalin and embedded in paraffin for immunohistochemistry. The other half was snap frozen in liquid nitrogen and stored at −80°C. At autopsy, the mice were also examined for distant organ metastases, and the number of metastatic foci in liver and spleen were counted.

### Immunolocalization of cyclin D1, VEGF, COX-2 and MMP-9 in tumor samples

Pancreatic tumor samples were fixed with paraformaldehyde and embedded in paraffin. After washing with PBS, the slides were blocked with protein block solution (Dako) for 20 minutes and incubated overnight with anti-human VEGF, COX-2, MMP-9, and cyclin D1 antibodies (1:50, 1:100, 1:200, 1:100, and 1:100, respectively). The expression of VEGF, COX-2, MMP-9, and cyclin D1 were evaluated using an immunohistochemical method described previously [45].

### Immunofluorescence staining

Frozen sections in slides were de-paraffinized and rehydrated through a graded alcohol series. Antigen retrieval was performed by boiling the slides for 20 minutes in antigen retrieval solution (10 mM sodium citrate, 0.05% Tween-20, pH 6.0). Slides were cooled for 30 minutes at room temperature, rinsed briefly in PBS, circled with a PAP pen and blocked with 1% normal goat serum. The sections were incubated with primary antibodies followed by their respective secondary antibodies (anti-rabbit IgG Alexa Fluor 610 and anti-mouse IgG Alexa Fluor 488). Slides were mounted using Vectashield Mounting Medium with DAPI (Vector Labs) viewed with a Nikon FXA fluorescence microscope equipped with Photometrics Cool Snap CF color camera (Nikon, Lewisville, TX) and MetaMorph version 4.6.5 software (Molecular Devices). The stored images were processed using NIH ImageJ software and Adobe Photoshop.

### Western blot analysis

Pancreatic cancer (Panc-28) cells and tumor tissues (75–100 mg per mouse) from vehicle control and experimental mice were minced and incubated, and Western blot analysis was performed, as previously described [45].

### Quantitative real time RT-PCR analysis

To examine the effects of UA, gemcitabine and the combination of two agents on miR-29a expression in tumor tissues, we performed quantitative real time PCR as described previously [47]. Total RNA isolated from the tumors of control and treated mice were reverse transcribed using Revert Aid reverse transcriptase following the manufacturer's protocol. The quantitative real time PCR reaction solution contained 5 μL of 2x SYBR green PCR master mix (Applied Biosystems), 3 μL of water, 0.5 μL of 5 μM universal primer, 1 μL of 5 μM forward primer (the mature miRNA sequence converted to DNA) and 1 μL of the diluted (4 times) cDNA. The internal control was derived from average levels of 5s RNA and RnuI. The data were analyzed by comparative CT method and the fold change was calculated by 2^−ΔΔCT^ method [48].

### Bioavailability analysis

The mice were fed UA 4 hours before being euthanized. The mice were anesthetized, blood was collected by cardiac puncture, and then the mice were killed humanely. High-performance liquid chromatography (HPLC) analysis was performed as previously described [29] to determine bioavailability of UA in the serum and the tumors.

### Statistical analysis

Data are presented as mean ± SD. The Student *t*-test was used to compare means of 2 independent variables. One-way analysis of variance was used to determine the statistical differences between more than 2 groups. Statistical significance was established as *P* < 0.05. A significant interaction was interpreted by a subsequent median effect principle of the Chou-Talalay method.

## SUPPLEMENTARY FIGURES


