# Supplementary material for: Ursolic acid inhibits the growth of human pancreatic cancer and enhances the antitumor potential of gemcitabine in an orthotopic mouse model through suppression of the inflammatory microenvironment
Source: Oncotarget. 2016 Feb 20;7(11):13182–96. doi: 10.18632/oncotarget.7537 (PMC4914350; doi:10.18632/oncotarget.7537)
Supplement: Supplementary file 1 [file oncotarget-07-13182-s001.pdf]

SUPPLEMENTARY FIGURES

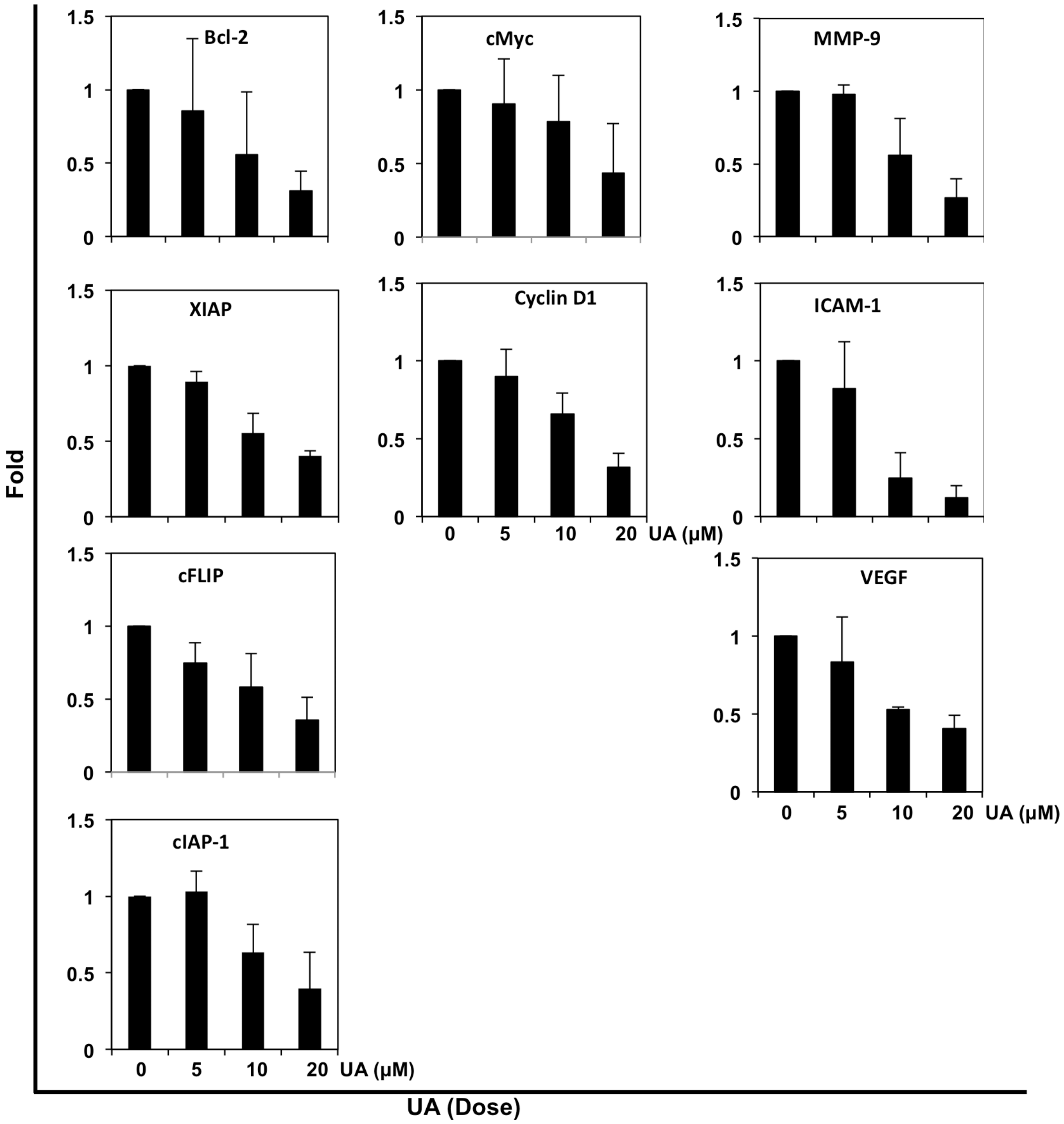

Supplementary Figure S1: Densitometric analysis of protein bands. Western blot was performed with cell extracts treated with different doses of UA. The density of protein bands was analyzed by using Image J software.

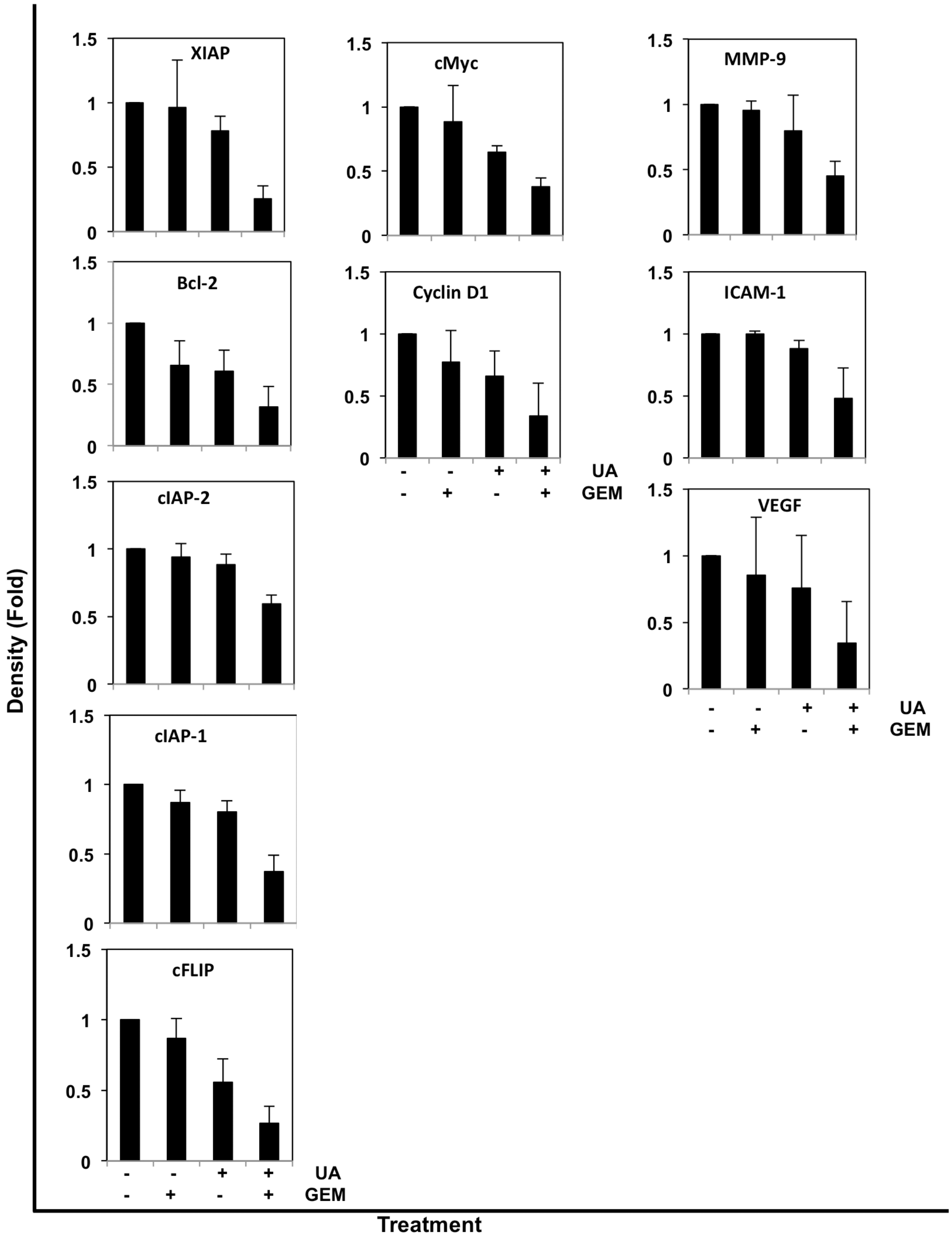

Supplementary Figure S2: Densitometric analysis of protein bands. Western blot was performed with cell extracts treated with UA and gemcitabine**.** The density of protein bands was analyzed by using Image J software.
